# Analgesic Effect of Preoperative Pentazocine for Laparoscopic Cholecystectomy

**DOI:** 10.7759/cureus.948

**Published:** 2016-12-31

**Authors:** Na Wang, Lei Wang, Yang Gao, Honglan Zhou, Jinguo Wang

**Affiliations:** 1 Department of Anesthesiology, The First Hospital of Jilin University; 2 Department of Cardiovascular Surgery, The First Hospital of Jilin University; 3 Department of Urology, The First Hospital of Jilin University; 4 Department of Urology, The First Hospital of Jilin University

**Keywords:** pentazocine, preemptive analgesia, laparoscopy, stress reaction, postoperative pain

## Abstract

Objective*:* To assess whether preoperative pentazocine can reduce intraoperative hemodynamic changes and postoperative pain.

Methods*:* Fifty patients undergoing laparoscopic cholecystectomy were randomized into two groups. Group P received intravenous 0.5 mg/kg pentazocine 10 min before surgery, and Group C received normal saline as a placebo. A standardized general anesthesia was conducted in all patients. Mean blood pressure (MBP), heart rate (HR), and visual analog scale (VAS) scores at various time points were recorded. The tramadol consumption during the study period was recorded.

Results: Group P had lower VAS scores at two, four, and eight hours postoperatively compared with Group C. MBP and HR rose significantly because of pneumoperitoneum within Group C, and no significant changes were detected in MBP and HR within Group P. Tramadol doses given were statistically fewer in Group P.

Conclusion: Preoperative intravenous pentazocine can decrease intraoperative hemodynamic changes and postoperative pain.

## Introduction

Although laparoscopic surgery is associated with less tissue damage than open surgery, intraoperative stress reaction and postoperative pain are still common. Preemptive analgesia is a pretreatment which prevents the establishment of central sensitization that can amplify the upcoming pain. Various drugs have been used for this analgesic method. Pentazocine, which acts as an agonist of Kappa receptors (κ receptors) and partial agonist/antagonist of M receptors, has been widely used to treat moderate to severe pain. It is recognized that pentazocine has an adequate analgesic efficacy with relatively weak respiratory depression and addiction [1-2].

We designed this clinical research to assess the analgesic efficacy of preoperative intravenous pentazocine for laparoscopic cholecystectomy under general anesthesia.

## Materials and methods

After approval of the Institutional Ethics Committee of the First Hospital of Jilin University (approval #2016-291) and obtaining written informed consent from the participants, this randomized and double-blinded study was conducted on 50 patients undergoing laparoscopic cholecystectomy under a standardized general anesthesia. Patients allergic to the study drugs, with American Society of Anesthesiologists (ASA) physical status more than two, a history of drug abuse, communication difficulties, and morbidly obese (body mass index > 30 kg/m^2^)  patients were excluded from this study.

Randomization was performed with a computer-generated sequence of numbers and sealed envelopes. Group P received 0.5 mg/kg pentazocine (pentazocine, CR Double-Crane Pharmaceuticals Co. Ltd, Beijing, China) 10 min before the surgery, and Group C received a placebo of normal saline. The study medications were prepared by a researcher who was not involved in the management of anesthesia. The other researchers and patients were blind to the grouping allocation. All patients were instructed on the visual analog scale (VAS) preoperatively and its use as a method for measuring postoperative pain.

After shifting the patients to the operating theatre, electrocardiogram (ECG), heart rate (HR), blood pressure (BP), Narcotrend index (NI), end-tidal carbon dioxide, and oxygen saturation (SpO_2_) were monitored. Normal saline was infused at the rate of 8∼10 ml/kg/h. Anesthetic induction was conducted using 0.03 mg/kg midazolam, 0.3 mg/kg etomidate, 4 µg/kg fentanyl, and 0.15 mg/kg cisatracurium. Anesthesia was maintained with 6 to 8 mg/kg/h propofol, 0.008 mg/kg/h remifentanil, and intermittent administration of cisatracurium as needed. Intravenous 1 µg/kg fentanyl was given to all patients about 10 min before the completion of the surgery.

Mean blood pressure (MBP) and HR were measured and recorded every five minutes. The pain intensity was assessed by a linear 10 cm visual analog scale (VAS: 0- no pain; 1, 2, 3 - mild pain; 4, 5, 6 - moderate pain; 7, 8, 9 - severe pain; 10 - worst imaginable pain); the sedation status was evaluated with the Ramsay sedation scale (RSS: 1 - anxious and agitated, 2 - cooperative and tranquil, 3 - drowsy but responds to command, 4 - asleep but responds to tactile stimulation, and 5 - asleep and no response). VAS scores at rest were evaluated and recorded at two, four, eight, 12, and 24 hrs after extubation of the patients. Postoperative analgesia consisted of 1.5 mg/kg tramadol, which was administrated intravenously when the patient complained of pain and the VAS score was more than 3. The time to the first analgesic request and tramadol doses given were recorded.

Adverse effects associated with pentazocine, such as nausea, vomiting, itching, oversedation, and respiratory depression were assessed with a “yes” or “no” survey. Oversedation was defined as respiratory severity score (RSS) equal to five. Respiratory depression was defined as respiratory rate less than 10 breaths/min or SpO_2_ less than 90.

The time to the first analgesic request was the primary endpoint of this study. According to our clinical experiences, we assumed that preoperative intravenous pentazocine would extend 30 min of this time period; 23 subjects were necessary for each group with two-sided α of 5% and β of 10%. Twenty-five patients were enrolled in each group for possible dropouts.

SPSS 17.0 (SPSS Inc, Chicago, IL, USA) was used for statistical analyses. Data were analyzed with unpaired t-test for normally distributed data, Mann–Whitney U-test for non-normally distributed data (the number of tramadol doses and VAS scores), and Chi-square or Fisher’s exact test for qualitative data. A p < 0.05 was regarded as statistically significant.

## Results

No significant difference was found between the two groups in demographic data and surgical characteristics (Table 1).

**Table 1:** Demographic Data and Surgical Characteristics Values are presented as mean ± standard deviation and number of patients.

	Age (yr)	Weight (kg)	Male/Female	ASA Ⅰ/Ⅱ	Duration of surgery (min)
Group P (n = 25)	45.2 ± 7.8	56.4 ± 7.2	11/14	19/6	26.7 ± 12.6
Group C (n = 25)	47.7 ± 8.4	54.8 ± 9.4	10/15	21/4	28.5 ± 11.4
P-value	0.281	0.502	1.000	0.725	0.598

**Figure 1:**
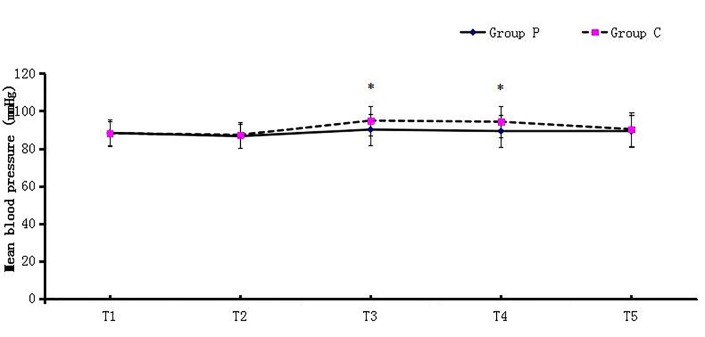
Mean Blood Pressure at Various Time Points Abbreviations: T1, the time of arrival at the operating room; T2, 5 min before skin incision; T3, T4, and T5, 5 min, 10 min, and 15 min after pneumoperitoneum, respectively. * indicates p* *< 0.05 between the two groups.

**Figure 2:**
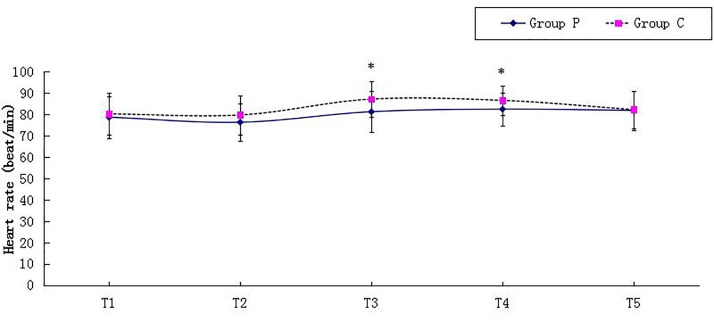
Heart Rate at Various Time Points Abbreviations: T1, the time of arrival at the operating room; T2, 5 min before skin incision; T3, T4, and T5, 5 min, 10 min, and 15 min after pneumoperitoneum, respectively. * indicates p* *< 0.05 between the two groups.

Figures 1 and 2 presented MBP and HR at 5 min after arrival at the operating room (T1), 5 min before surgery (T2), 5 min (T3), 10 min (T4), and 15 min (T5) after pneumoperitoneum. In Group C, MBP at T3 and T4 was significantly higher than MBP at T1 and T2; HR at T3 was higher compared with T1 and HR at T4 was higher compared with T1 and T2 within Group C because of pneumoperitoneum. Significant differences were detected in MBP and HR at T3 and T4 between the two groups, and MBP and HR were higher in Group C (Figures 1-2).

**Figure 3:**
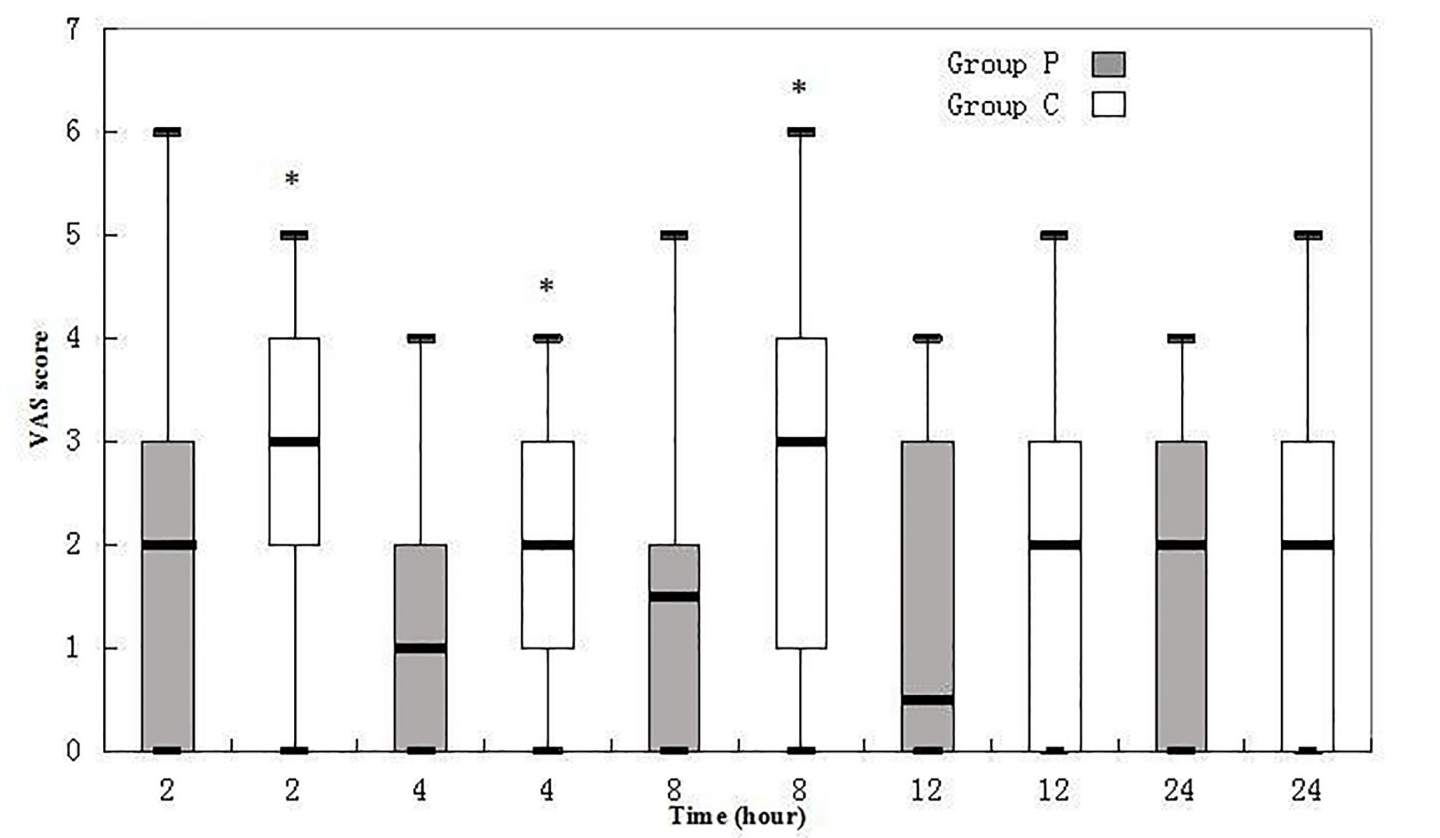
Box Plots of VAS Scores at Various Time Points Results are expressed in the median. The top and bottom of each box indicate 75th and 25th percentiles and the error bars maximum and minimum values. VAS: visual analogue scale. * indicates p* *< 0.05 compared with the counterpart of Group P.

VAS pain scores at two, four, and eight hrs after surgery were significantly lower in Group P (Figure 3).

The incidence of side effects was comparable between the two groups during the study period. Tramadol doses given were statistically lower in Group P than in Group C (P *= *0.033) (Table 2).

**Table 2:** Side Effects and Tramadol Consumption Values are presented as mean ± standard deviation and number of patients.

	Nausea	Vomiting	Itching	Oversedation	Tramadol consumption (dose)
Group P (n = 25)	4 (16%)	1 (4%)	3 (12%)	1 (4%)	0.84 ± 0.85
Group C (n = 25)	3 (12%)	1 (4%)	2 (8%)	0 (0%)	1.36 ± 0.81
P-value	1.000	1.000	1.000	1.000	0.033

No respiratory depression was observed in either group.

## Discussion

The present study demonstrates that intravenous pentazocine administrated 10 min before surgery provides a reduction in intraoperative hemodynamic change, postoperative pain intensity, and tramadol consumption. Although laparoscopic cholecystectomy is associated with less tissue damage, port pain, abdominal organ nociception, and diaphragmatic irritation from residual pneumoperitoneum contribute to the total pain intensity perceived [3-5]. Therefore, some patients still suffer significant postoperative pain. Administrating an analgesic medication before the start of the surgical stimuli can decrease or block the development of central pain sensitization and then result in less postoperative pain [6].

MBP and HR are studied as an indirect reflection of intraoperative stress reaction. Patients in Group C had higher MBP and HR after pneumoperitoneum and during exploration of upper abdominal organs. This indicates that patients in Group C had a stronger stress reaction to surgical stimuli. The lower MBP and HR in Group P may contribute to the preemptive analgesic effect of pentazocine.

Pentazocine is a synthetically mixed agonist–antagonist narcotic (opioid analgesic) drug used to treat moderate to severe pain. The advantage of pentazocine is that it is a morphine antagonist and has not yet been found to produce tolerance or dependence [7]. Bao, et al. find that activating opioid receptors in the dorsal root ganglion causes intracellular Ca^2+^ release and Ca^2+^ entry, which leads to a release of excitatory neuropeptides [8]. It suggests that the preemptive effect of pentazocine may result from the blockade of opioid receptors. The specific mechanism is unclear.

No literature has been found about preemptive analgesia with pentazocine. Because the peak time of intravenous pentazocine is 2∼3 min, 10 min prior to surgery was chosen as the time of preemptive analgesia in this clinical trial [9].

The incidences of these side effects were comparable between the two groups. No respiratory depression was observed in this study. The result is in line with the previous studies that pentazocine has relatively weak respiratory disturbances [1-2].

One limitation of our study is that laparoscopic cholecystectomy is a relatively small surgery, so the result of this study is limited and cannot be expanded to major surgeries. Future research will be conducted on major surgeries. There are many analgesic modes and ours is a simple one, so the analgesic method of this study may not be suitable for other groups.

## Conclusions

Preoperative pentazocine can decrease hemodynamic changes, postoperative pain, and analgesic requirement in patients undergoing laparoscopic cholecystectomy. 
